# Musashi1 modulates cell proliferation genes in the medulloblastoma cell line Daoy

**DOI:** 10.1186/1471-2407-8-280

**Published:** 2008-09-30

**Authors:** Patricia C Sanchez-Diaz, Tarea L Burton, Suzanne C Burns, Jaclyn Y Hung, Luiz OF Penalva

**Affiliations:** 1 Greehey Children's Cancer Research Institute, University of Texas Health Science Center at San Antonio, TX, USA; 2 Division of Hematology and Oncology, Department of Pediatrics, University of Texas Health Science Center at San Antonio, TX, USA; 3 Department of Cell and Structural Biology, University of Texas Health Science Center at San Antonio, TX, USA

## Abstract

**Background:**

Musashi1 (Msi1) is an RNA binding protein with a central role during nervous system development and stem cell maintenance. High levels of Msi1 have been reported in several malignancies including brain tumors thereby associating Msi1 and cancer.

**Methods:**

We used the human medulloblastoma cell line Daoy as model system in this study to knock down the expression of Msi1 and determine the effects upon soft agar growth and neurophere formation. Quantitative RT-PCR was conducted to evaluate the expression of cell proliferation, differentiation and survival genes in Msi1 depleted Daoy cells.

**Results:**

We observed that *MSI1 *expression was elevated in Daoy cells cultured as neurospheres compared to those grown as monolayer. These data indicated that Msi1 might be involved in regulating proliferation in cancer cells. Here we show that shRNA mediated Msi1 depletion in Daoy cells notably impaired their ability to form colonies in soft agar and to grow as neurospheres in culture. Moreover, differential expression of a group of Notch, Hedgehog and Wnt pathway related genes including *MYCN*, *FOS*, *NOTCH2*, *SMO*, *CDKN1A*, *CCND2*, *CCND1*, and *DKK1*, was also found in the Msi1 knockdown, demonstrating that Msi1 modulated the expression of a subset of cell proliferation, differentiation and survival genes in Daoy.

**Conclusion:**

Our data suggested that Msi1 may promote cancer cell proliferation and survival as its loss seems to have a detrimental effect in the maintenance of medulloblastoma cancer cells. In this regard, Msi1 might be a positive regulator of tumor progression and a potential target for therapy.

## Background

Musasi1 (Msi1) is an RNA binding protein essential during nervous system development. It is considered a stem cell marker whose expression has been found to be conserved across species from fly to human [1]. In the mammalian postnatal brain, Msi1 is mainly expressed in cells that are believed to be the source of adult neural stem cells [2] and seems to be critical for their maintenance and self-renewal capability [1,3,4].

High levels of Msi1 have been reported in tumors such as medulloblastoma [5,6], glioma [7,8], astrocytoma [9], retinoblastoma [10] and colorectal adenoma [11]. Indeed, a correlation between high levels of Msi1 expression and poor prognosis has been proposed for glioma and astrocytoma [8,9].

Two Msi1 direct targets have been characterized in mammals: *numb *[12] and *CDKN1A *[13]. Binding of Msi1 to specific motifs located in the 3' untranslated region (UTR) of these mRNAs seems to interfere with translation, thereby decreasing Numb and p21^WAF ^(also known as Cdkn1a) protein levels [12,13]. Numb is a regulator of three important pathways usually deregulated in cancer: Notch, Hedgehog and p53 (reviewed in [14-17]). Numb represses Notch [18] and Hedgehog [19]. In addition, Numb has recently been shown to prevent degradation of the tumor suppressor p53 [20]. The second known target of Msi1 is the cell cycle inhibitor p21^WAF^. Therefore, it is plausible to surmise that by repressing translation of Numb and p21^WAF^, high levels of Msi1 might promote aberrant cell proliferation and failure in differentiation and apoptosis.

We observed that the levels of *MSI1 *were elevated in Daoy neurospheres (high proliferative cultures) compared to monolayers (low proliferative cultures). This data suggested a potential role for Msi1 in promoting cancer cell proliferation in this medulloblastoma cell line. In order to test this hypothesis, we depleted Msi1 in Daoy cells by RNA interference. A significant reduction in soft agar growth (*in vitro *indicator of tumorigenicity) and neurosphere formation (surrogative measure of "stemness") were observed. We also identified a set of cell proliferation genes whose expression was significantly down-regulated after Msi1 shRNA-mediated knockdown. Thus our data suggested that Msi1 may promote cancer cell proliferation. We propose that Msi1 may maintain a pool of cancer cells with deregulated proliferative capabilities which may possibly serve as a source for future tumorigenic events. In this regard, Msi1 might be a positive regulator of tumor progression and a prospective target for therapeutic intervention.

## Methods

### Cell lines, plasmids and transfections

Daoy cell line was obtained from American Type Culture Collection (ATCC). Cells were cultured in improved minimum essential medium (IMEM) (Invitrogen, Carlsbad, CA, USA) supplemented with 10% fetal bovine serum (Atlanta Biologicals, Inc., Lawrenceville, GA, USA). Msi1 was knocked down using a shRNAmir retroviral vector targeting the sequence 5'-CGTCCTGTATCATATGTAAAT-3' located in the 3'UTR of Msi1 mRNA (Oligo ID # V2HS_280120; Open Biosystems). Cells were transfected at 95% confluency using Lipofectamine2000 reagent (Invitrogen) according to manufacturer's instructions. Stable integration of the plasmid encoding the shRNA was selected using 1 μg/mL of puromycin (InvivoGen, San Diego, CA, USA). A stable Daoy cell line expressing a non-silencing shRNAmir (Open Biosystems) was also generated as a negative control.

### Musashi1 polyclonal antibody generation

A 174 nucleotide sequence encoding a 65 aminoacid peptide unique for Msi1 (FPEFRVERTPLPSAPVLPELTAIPLTAYGPMAAAAAAAAVVRGTGSHPWTMAPPPGSTLERPHRD) was cloned into pGEX-4T-1 (GE Healthcare, Piscataway, NJ, USA) to generate a GST-Msi1 fusion protein. GST-Msi1 recombinant protein was purified to >90% homogeneity using Glutathione Sepharose 4B (GE Healthcare) according to manufacturer's instructions. Antibodies raised against Msi1 were affinity-purified from the rabbit antisera by column chromatography in two steps: first using the antigen (GST-Msi1 protein) and then using the GST purified protein. The antibodies were eluted in PBS containing 0.01% of sodium azide and stored at -80°C.

### Western blotting

For western blot analysis cells were disrupted in lysis buffer as described [45]. Equal amounts of protein extract (50 μg per lane) were solubilized in 2× reducing sample buffer (62.5 mM Tris-Hcl pH 6.8, 25% glycerol, 2% SDS, 0.1% w/v Orange G and 40 mM DTT), run on 12% Tris-glycine SDS-Polyacrylamide gels and transferred to nitrocellulose membranes (Invitrogen) using a semi-dry trans-blot system (Bio-Rad Laboratories, Inc., Hercules, CA, USA). Membranes were stained with 0.1% w/v Ponceau Red (Sigma-Aldrich, St Louis, MO, USA) solution, blocked in 0.1% Tween 20/PBS containing 5% w/v non-fat dry milk and incubated over night at 4°C with the appropriated antibody; anti-βIII Tubulin (Abcam, Inc., Cambridge, MA, USA), anti-Bcl-2 (Santa Cruz Biotechnology, Inc., Santa Cruz, CA, USA), anti-β-Actin (loading control; Abcam) following manufacturers' guidelines, or rabbit anti-Musashi1 polyclonal antibody (described above) at a 0.5 μg/mL final concentration in blocking buffer. After washing in 0.1% Tween 20/PBS, blots were incubated for 1 hour at room temperature with goat anti-rabbit IgG HRP conjugated secondary antibody (Santa Cruz) and developed using enhanced chemiluminescence detection (Pierce Biotechnology, Rockford, IL, USA). Experiments were performed twice using different cell extracts. Band densitometry was performed using Adobe Photoshop CS3 Extended version 10.0 software (Adobe Systems Incorporated).

### Colony formation assay

The ability of Daoy cells to grow in soft agar was analyzed as described [21]. Briefly, 60 mm soft agar plates containing 10% FBS (Atlanta Biologicals) MEM (Invitrogen) and 0.4% agar noble (BD Biosciences, San Jose, CA, USA) were inoculated with a suspension of 3 × 10^3 ^cells of either control or Msi1 knockdown Daoy. Cells were fed once a week by adding 0.5 ml of complete IMEM medium (Invitrogen). After 8 weeks of incubation, colonies were stained over night with 1 mg/mL *p*-iodonitrotetrazolium solution (Sigma) and scored using the colony counting application from the Quantity One software (Bio-Rad). Three individual clones were tested with each sample analyzed in quadruplicate. Two independent experiments were performed with similar results.

### Neurosphere culture assay

Daoy neurosphere culture was performed as described [46] Briefly, cells were trypsinized and washed in Neurobasal medium (Invitrogen) and resuspended at 5 × 10^4 ^cells/mL in the same medium containing 2 mM L-glutamine, N2 supplement, B27 supplement, 20 ng/mL hrEGF, 20 ng/mL hrbFGF and 50 μg/mL BSA (Invitrogen). 1 μg/mL of puromycin (InvivoGen) was included to ensure shRNA plasmid selection. Fresh growth factors were added to the cells twice a week. Neurospheres were disaggregated in single-cell suspensions and reseeded at clonal density as described [46] to form secondary, tertiary and quaternary spheres. For the neurosphere dilution assay single-cell suspensions of control and Msi1 knockdown Daoy cells were plated in Neurobasal medium in 96-well plates (serial dilutions from 1000 to 1 cell/well). After 10 days incubation the number of spheres larger than 50 μm in diameter were quantified in 8 wells. Two independent experiments were performed with similar results.

### Quantitative RT-PCR

RNA was prepared using Trizol reagent (Invitrogen), treated with RNase-free DNaseI (Roche Diagnostics, Indianapolis, IN, USA) as per manufacturer's protocols and tested by PCR to ensure the absence of genomic DNA contamination. Gene specific primers for real-time RT-PCR, listed in Additional file 1, were designed using Primer3 [47] and purchased from Integrated DNA Technologies (IDT, Coralville, IA, USA). One-step RT-PCR reactions were performed using an ABI 7500 real time PCR system (Applied Biosystems, Foster City, CA, USA) following manufacturer's instructions. Briefly, 50 ng total RNA was reverse transcribed at 48°C for 45 min using 150 nM gene specific primers, 0.5 U/μL of MultiScribe Reverse Transcriptase and 0.4 U/μL of RNase Inhibitor in 1× SYBR Green PCR Power Master Mix (ABI). RT enzyme was heat inactivated at 95°C for 10 min and PCR was carried out as follows: One cycle of 95°C for 5 min and 40 cycles of 15 s at 95°C followed by 1 min at 50–60°C according to primers' Tm. Relative gene expression was determined using the comparative C_T _method and the ABI 7500 Prism Software. *15S *RNA levels were used as endogenous control for normalization. Specificity of PCR amplification was confirmed by the dissociation curves of the amplicons. Samples were analyzed in quadruplicate using two different RNA preparations.

### Hedgehog pathway blockade

Cyclopamine and its analog tomatidine were purchased from Calbiochem (San Diego, CA, USA) and stocked as 5 mM solution in 95% ethanol. Both drugs were used at a concentration range of 5–20 μM in Neurobasal medium prepared as described above. Briefly, neurospheres were dissociated as described [46] and single-cell suspensions reseeded at clonal density in 12-well plates (10,000 cells/well). The effects of Hedgehog blockade upon sphere formation in Daoy control and knockdown cells were evaluated after 7 days of incubation. Two experiments were performed with similar results.

### Statistical analysis

Statistical analysis was performed using GraphPad (GraphPad Software, Inc., San Diego, CA, USA). Data from quantitative RT-PCR, soft agar growth and SP is presented as means ± standard error (se), and were analyzed by two-tailed unpaired Student's t-test. Differences were considered significant when P-value was below 0.05.

## Results

### Msi1 expression in Daoy neurosphere cultures

**Figure 1:**
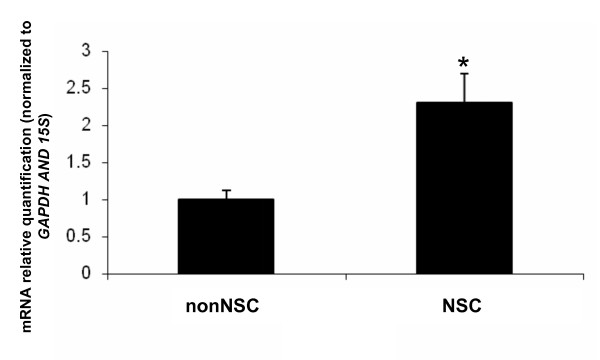
**Msi1 expression in Daoy neurospheres**. The levels of *MSI1 *RNA levels were analyzed in Daoy cells grown as monolayer (nonNSC) and as neurosphere (NSC) cultures using quantitative RT-PCR. *GAPDH *and *15S *mRNA levels were used as endogenous control for normalization. On average, a 2-fold increase in *MSI1 *was detected in the neurosphere cultures.* indicate p < 0.01.

To determine if Msi1 might play a role in Daoy cancer cell proliferation, we measured the levels of *MSI1 *mRNA in Daoy neurosphere cultures by quantitative RT-PCR (Figure 1). On average, a 2-fold increase in MSI1 level was detected in neurospheres (actively proliferating culture) compared to monolayers (less actively proliferating culture). Since neurosphere cultures can be enriched in tumor re-initiating cells and Msi1 is a stem cell marker with a role in cell cycle progression [13], the higher level of Msi1 detected in neurospheres indicated that Msi1 may contribute to Daoy cancer cell proliferation.

### Generation of a Msi1 knockdown in the medulloblastoma cell line Daoy

We depleted Msi1 in the medulloblastoma cell line Daoy using a vector-based siRNA which targeted a sequence unique for Msi1 located in the 3'UTR of the gene. As a negative control, cells were transfected with the same vector containing a non silencing shRNA (Open Biosystems, Inc., Huntsville AL, USA). Three individual clones exhibiting at least a 70% knockdown efficiency at the mRNA level (determined by quantitative RT-PCR; Additional file 1) were selected. Msi1 protein knockdown was confirmed by western blot (Additional file 1) using a polyclonal antibody generated in our laboratory.

### Msi1 knockdown impaired anchorage-independent growth in Daoy cell line

**Figure 2:**
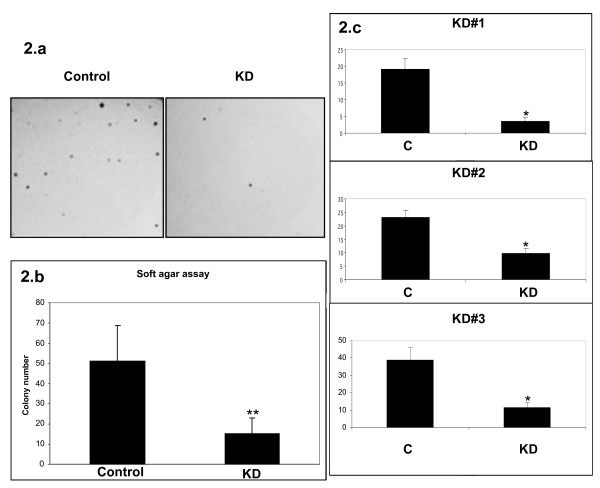
**Anchorage-independent growth**. The effect of Msi1 expression upon anchorage-independent growth was studied as an *in vitro *indicator of tumorigenicity in Daoy cells. Two independent experiments were performed with similar results. Msi1 depletion reduced the ability to form colonies in soft agar by 3 to 4-fold which indicated that Msi1 may sustain the malignant potential of this tumor cell line. a) A representative image of the colonies formed by the control and knockdown (KD) cell lines is shown. The average results for the three clones (b) and for each individual one (c) are represented. * and ** indicate p < 10^-3 ^and p < 10^-5^, respectively, compared to control.

We analyzed the effect of Msi1 on anchorage-independent growth (clonogenicity) in Daoy cells as an *in vitro *assay for tumorigenicity. Colony formation assays were performed in soft agar as previously described [21]. As shown in Figure 2, Msi1 knockdown reduced the ability of Daoy cells to form colonies in soft agar by 3 to 4-fold thus indicating that Msi1 promoted cell proliferation in these cancer cells and, consequently, that might play a role in tumor growth.

### Msi1 sustained cancer cells

**Figure 3:**
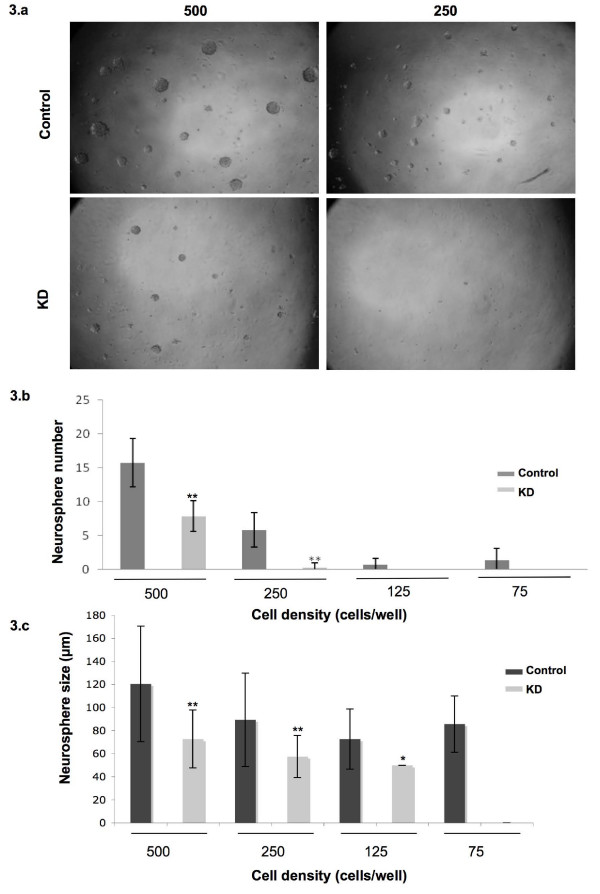
**Neurosphere formation assay**. a) Second generation of neurospheres derived from Daoy control (upper) and knockdown (KD; lower) at two different cell densities (500 and 250 cells/well) are shown. The smaller size neurospheres detected in the knockdown suggested that Msi1 controls cell proliferation in Daoy. b) The average number of spheres per well is represented at different cell densities (500, 250, 125 and 75 cells/well). Only spheres larger than 50 μm in diameter were scored. c) The differences in neurosphere size are represented at different cell densities (500, 250, 125 and 75 cells/well). * indicates p < 0.05 and ** p < 0.0001.

Msi1 is a stem cell marker reported to be essential for maintenance of stem cells [1,3,4]. Cells with stem-like properties have been isolated in many human brain cancers including medulloblastoma (reviewed in [22]). Since high levels of Msi1 are frequently found in medulloblastoma and since we observed that Msi1 expression was elevated in Daoy neurosphere compared to monolayer cultures (Figure 1), we asked if Msi1 might be involved in regulating proliferation in cancer cells. Here we evaluated neurosphere formation as an indicator of cell proliferative potential. Single-cell suspensions were plated at clonal density to minimize the effects of cell aggregation in favor of single-cell sphere generation. After 10 days of incubation, neurospheres larger than 50 μm in diameter were counted. As shown in Figure 3, a significant reduction in both number and overall size of the spheres was detected when Msi1 expression was depleted. The defect observed in sphere formation was more severe in subsequent serial passages (data not shown), thus indicating a role for Msi1 in sustaining cancer cells.

### Msi1 regulates expression of Notch, Hedgehog and Wnt components

**Figure 4:**
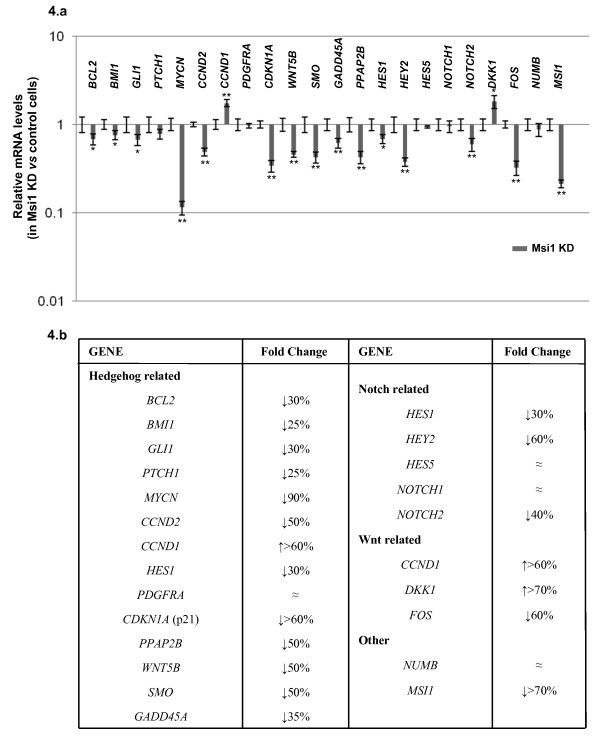
**Msi1 downstream effects**. The RNA levels of a subset of Notch, Hedgehog and Wnt pathway components were quantified by quantitative RT-PCR in control and knockdown Daoy cells. Gene expression changes are represented (mean ± se) using semi-logarithmic scale. The plot in the upper part (a) shows the fold change in mRNA levels for the Msi1 knockdown normalized to the housekeeping gene *15S*. The table (b) summarizes the ratio (in percentage) of mRNA levels detected in Msi1 knockdown vs control. * represents p < 0.05 and ** p < 0.01.

Msi1 represses translation of *NUMB *[12], which antagonizes the activity of Notch and Hedgehog [18,19]. Recently, it has been shown that Msi1 also activates Notch and Wnt in mammary progenitors through an autocrine mechanism involving up-regulation of Proliferin-1 and down-regulation of Dickkopf-3 [23]. Notch, Hedgehog and Wnt pathways are central regulators of cell proliferation and their deregulation is frequently associated with medulloblastoma (reviewed in [22]). With the high levels of Msi1 reported for medulloblastoma, a connection among Msi1, Notch, Hedgehog and Wnt activities and tumor growth is likely. To investigate if Msi1 was modulating the activity of these pathways in Daoy, the expression of some Notch, Hedgehog and Wnt pathway components and downstream targets were evaluated by quantitative RT-PCR. The primers used for this analysis are listed in Additional file 1. In the knockdown, up-regulation of the Wnt inhibitor Dickkopf-1 (*DKK1*; >70% increase) was observed along with a down-regulation in the Wnt downstream target *FOS *(60% reduction) as shown in Figure 4. Down-regulation of a subset of Notch and Hedgehog related genes was also found. In this regard, lower levels of the Hedgehog component *SMO *(50% decrease) and of the downstream targets *MYCN *(90% decrease), *CCND2*, *PPAP2B *and *CDKN1A *(50% decrease) were detected in the Msi1 knockdown cells together with lower levels of the relevant Notch component *NOTCH2 *(40% decrease) and of the downstream target *HEY2 *(60% decrease). Intriguingly, up-regulation of the Wnt and Hedgehog common downstream target *CCND1 *(>60% increase) was observed. Overall it seemed that Msi1 modulates expression of a number of genes involved in cell proliferation, differentiation and survival processes. Therefore, our observation was in agreement with Wang *et al *recent findings using mammary progenitors [23].

### Msi1 knockdown potentiated the effects of Hedgehog blockade

**Figure 5:**
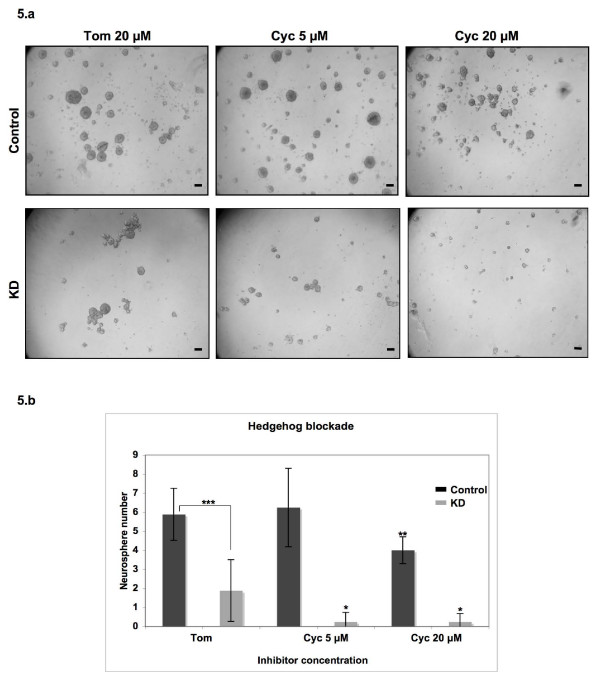
**Effect of Hedgehog blockade upon neurosphere formation**. The ability to form neuropheres in the presence of the Hedgehog inhibitor cyclopamine was evaluated in Daoy cells. Spheres derived from Daoy control (upper) and Msi1 knockdown (KD; lower) were incubated for one week in the presence of inhibitor (Cyc) and tomatidine (Tom; negative control) at a concentration range of 5–20 μM. A reduction in neurosphere size was observed in both cell lines; however, the knockdown cell line appeared to be more sensitive to cyclopamine treatment as the effects upon sphere formation were significant (p < 0.05) at lower concentrations of inhibitor. a) Representative images of the effects upon neurosphere formation at 5 μM and 20 μM cyclopamine are shown. Scale bars represent 100 μm. b) The average number of neurospheres per field is represented for control and KD cells after treatment with tomatidine and cyclopamine. * indicates p < 0.05, ** p < 0.01 and *** p < 0.001.

To determine the relevance of Hedgehog in cell proliferation in our model system, we performed a neurosphere assay in the presence of different concentrations of cyclopamine. Cyclopamine is a plant alkaloid which antagonizes Smoothened (Smo), a major signal transducer of the Hedgehog pathway [24]. As a negative control, we used the structural analog tomatidine at the same concentrations. While 5 μM cyclopamine induced a marked reduction in neurosphere size in the Msi1 knockdown cells (p < 0.05), 4 times higher concentrations were needed to elicit a significant effect in the control Daoy cells (p < 0.01; Figure 5). Therefore, Hedgehog seems to be important for neurosphere formation in Daoy cells, and the increased susceptibility to cyclopamine observed for Msi1 KD cells may reflect a partial down-regulation in the activity of this pathway.

### Msi1 potential implication in cell differentiation and apoptosis

**Figure 6:**
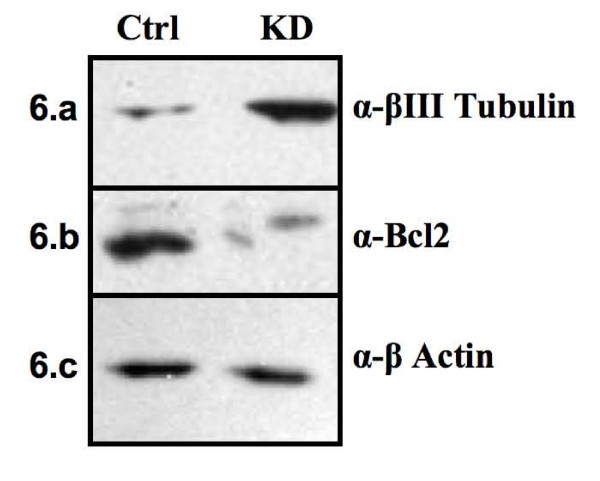
**Cell differentiation and apoptosis**. The levels of the neural marker βIII Tubulin and of the antiapoptotic protein Bcl2 were analyzed by western blot. The experiment was performed twice using two different cell extracts. a) The higher level of βIII Tubulin detected in knockdown (KD) *vs *control (Ctrl) indicates that Msi1 may play a role in inhibiting cell differentiation. In addition, the reduced levels of Bcl2 detected in the knockdown (b) suggested a potential role for Msi1 in preventing apoptosis. c) β-Actin was used as loading control.

In order to determine Msi1's possible involvement in cell differentiation and apoptosis we analyzed the levels of the neural marker βIII Tubulin and the antiapoptotic protein Bcl2 by western blot in the cell line Daoy (Figure 6). On average, a 5-fold increase (p < 0.05) in βIII Tubulin and a 8-fold decrease (p < 0.05) in Bcl-2 were detected when Msi1 was knocked down. The higher levels of βIII Tubulin detected in the knockdown indicated that Msi1 arrests cell differentiation. Decline in both Bcl2 protein (Figure 6) and *BCL2 *transcript (Figure 4) levels were also detected in the knockdown suggesting that Msi1 may block apoptosis in Daoy cells. As a more important reduction was detected at protein level than at mRNA level (~30% down-regulation; Figure 4), additional post-trancriptional mechanisms might be mediating the decrease of Bcl2 protein in our model system. Overall, the results obtained in our model system are consistent with a recent report by Dobson *et al*. [25] which demonstrates that Msi1 has an inhibitory effect upon oligodendrocyte precursor differentiation and apoptosis.

## Discussion

Msi1 is an RNA binding protein with an essential role in maintaining the stem cell state [1,3,4]. High levels of Msi1 have been found in several malignancies, including medulloblastoma [5,6]. As Msi1 expression was elevated in Daoy neurospheres (Figure 1), we asked if Msi1 may promote expansion of cancer cells. In this study, we used the cell line Daoy as our model system. Daoy is a well-characterized cell line, which among other features, expresses elevated levels of Msi1 [5], is able to form colonies in soft agar and neurospheres when cultured in the presence of growth factors, has functional Notch, Hedgehog and Wnt signaling pathways and is tumorigenic in *nude *mice [26-28]. We performed a shRNA mediated knockdown of Msi1 in Daoy and found that cancer cells expressing low levels of Msi1 were less clonogenic (proliferation assay), more differentiated (higher βIII Tubulin expression) and, possibly, more apoptotic (lower Bcl2 expression). We also observed a defect in neurosphere formation which was consistent with a potential role of Msi1 in activating proliferation and/or preventing differentiation and apoptosis in Daoy cells. Taken together, our data suggest that Msi1 may contribute to the proliferation of cancer cells. This observation was in agreement with previous reports correlating high levels of Msi1 with glioma and astrocytoma malignancy [8,9].

Notch, Hedgehog, Wnt and p53 are important pathways that are frequently found to be deregulated in cancer (reviewed in [14-17]). Msi1 represses translation of *numb *[12]; an antagonist of Notch and Hedgehog signaling pathways [18,19] which also prevents degradation of the tumor suppressor p53 [20]. Msi1 also activates Wnt pathway through an autocrine mechanism [23]. Thus it is possible that high levels of Msi1 may deregulate Notch, Hedgehog, Wnt and p53 activities in cancer cells thereby contributing to tumor growth.

We performed gene expression analysis to determine the downstream effects derived from Msi1 depletion. Since Daoy is p53 defective, the effects detected in cell proliferation in our model system are therefore independent of p53. However, given that p53 inactivation is found in 50% of the malignancies (reviewed in [17]), an in-depth work to elucidate the possible effects of Msi1 upon p53 activity in cancer cells is highly desirable.

We demonstrated that Msi1 regulates the expression of several genes involved in cell proliferation and tumorigenicity such us *MYCN*, *SMO*, *NOTCH2*, *CCND1*, *CCND2*, *CDKN1A*, *FOS*, *GLI1*, and *DKK1 *[26,29-36]. Noteworthy was the differential expression of two cell cycle regulators: *CDKN1A *(encoding p21^WAF^) and *CCND1 *(encoding Cyclin D1). A 2-fold decrease was found in *CDKN1A *levels when Msi1 was depleted in Daoy, possibly due to a reduced Hedgehog activity. Battelli *et al*. [13] reported that over-expression of Msi1 in HEK293 activates cell proliferation by down-regulating p21^WAF ^protein levels. Using a knockdown model in Daoy, we showed that Msi1 increases p21^WAF ^transcription, possibly, *via *Hedgehog activation. Although found in different model systems, it seems that Msi1 may play a dual role in regulating p21^WAF^; positive and indirect at the level of transcription (as shown here) and negative and direct at the post-transcriptional level (as previously reported [13]).

The up-regulation of *CCND1 *detected in the Msi1 knockdown was intriguing. *CCND1 *transcription is induced in response to many oncogenic signals like Ras, ErB2, Src and Wnt (reviewed in [37]) and CyclinD1 overexpression is associated with tumorigenesis and metastasis. Moreover, *CCND1 *expression is considered an early event in malignancy and its loss inhibits tumor formation in a *Ptch1*^+/- ^mouse model [38]. On the other hand and in agreement with our observation, down-regulation of *CCND1 *has recently been reported by Rubio et al. during spontaneous mesenchymal cell transformation [39]. Therefore, the mechanism driving up-regulation of *CCND1 *and its biological implications in our knockdown model system would justify a more intense investigation.

Cross-talk within Notch, Hedgehog and Wnt pathways has often been reported [5,23,26,31,40-42] indicating that the activity of these pathways needs to be fine-tuned in order to maintain homeostasis. We observed that Msi1 regulated the expression of Hedgehog key components, including the pathway mediators *SMO *and *GLI1 *and the pathway downstream target *MYCN*, known to control cell proliferation and tumorigenicity [29,30,34]. Interestingly, simultaneous expression of *MSI1 *and *MYCN *occurs in clinical specimens , which posits *MYCN *as an attractive Msi1 downstream effector. Moreover, *MYCN *represses transcription of the tumor suppressor *DKK1 *[43]. We thus propose that down-regulation of *MYCN *in Daoy cancer cells after Msi1 depletion might induce expression of the Wnt repressor *DKK1*, therefore interconnecting Msi1, Wnt and Hedgehog signaling pathways. At least in part, this Msi1/MycN/Dkk-1 axis may explain the growth suppressive effect observed in our knockdown model system.

To determine the relevance of the Hedgehog pathway in Daoy cancer cells, we performed a neurosphere culture assay in the presence of cyclopamine. Cyclopamine inhibited neurosphere formation in both cases; however, the effects in the knockdown cells were evident at a significantly lower concentration of antagonist. This data was consistent with a partial down-regulation of Hedgehog activity after Msi1 depletion in Daoy cells. On the other hand, it does not exclude the possibility that Msi1 may also modulate the activity of additional cell proliferation pathways such as Notch or Wnt.

We also found that Msi1 increased *NOTCH2 *levels thus suggesting a potential role of Msi1 in contributing to tumor growth. *NOTCH2 *is a component of the Notch pathway that acts as a mitogen for cerebellar granule cell precursors [44] and whose expression has been associated with malignancy in brain tumors and with the persistence of cancer cells [26,27,33]. Indeed, it has been shown that Notch blockade reduces cancer cells in Daoy and inhibits tumor engraftment in nude mice [26,27].

## Conclusion

In summary, our data suggest that Msi1 modulates proliferation of cancer cells. Msi1 regulated expression of a set of genes with a well-established role in cell proliferation, cell differentiation and survival and its loss appeared to have a detrimental effect on the maintenance of cancer cells. We thus propose that, by hijacking normal cell signaling pathways, Msi1 may sustain cancer cells. Likely, Msi1 regulates additional genes and a systems biology approach needs to be carried out to identify new Msi1 direct targets (i.e. post-transcriptionally regulated genes) and uncover the gene network controlled by Msi1. We believe that the identification of additional Msi1 direct target genes is necessary to better understand the function of this RNA binding protein in cancer cells. These insights may provide a foundation for designing novel and more rational therapies.

## Competing interests

The authors declare that they have no competing interests.

## Authors' contributions

PCSD performed all the experiments, interpreted the results, contributed with scientific discussion and prepared the manuscript. TLB and SCB provided technical assistance. JYH and LOFP contributed with scientific discussion and manuscript preparation. All authors read and approved the final manuscript.

## Acknowledgements

We thank Susan L. Naylor for the assistance with soft agar assays and Raymond L. Stallings for his comments about the manuscript. Msi1 polyclonal antibody was generated at the San Antonio Cancer Institute (SACI) Antigen & Antibody core facility. This work was supported by the San Antonio Area Foundation (PGID 122760) and SACI and American Cancer Society (PGID 124139).

## Pre-publication history

The pre-publication history for this paper can be accessed here:



## Supplementary Material

Additional file 1 Table 1. qRT-PCR primers. The sequences of the primers utilized in this study are shown in the table. Click here for file
