# Supplementary material for: Musashi1 modulates cell proliferation genes in the medulloblastoma cell line Daoy
Source: BMC Cancer. 2008 Sep 30;8:280. doi: 10.1186/1471-2407-8-280 (PMC2572071; doi:10.1186/1471-2407-8-280)
Supplement: Additional file 1 — Table 1. qRT-PCR primers. The sequences of the primers utilized in this study are shown in the table. [file 1471-2407-8-280-S1.pdf]

1.1. Table 1. qPCR primers

Gene	***Forward (5'→3')***	***Reverse (5'→3')***
BCL2	TACCTGAACCGGCACCTG	GCCGTACAGTTCCACAAAGG
BMI1	TCATCCTTCTGCTGATGCTG	GCATCACAGTCATTGCTGCT
CCND1	GAACAAACAGATCATCCGCAAAC	GCGGTAGTAGGACAGGAAGTTG
CCND2	TGGGGAAGTTGAAGTGGAAC	ATCCACGTCTGTGTTGGTGA
CDKN1A (p21)	GGAAGACCATGTGGACCTGT	GGATTAGGGCTTCCTCTTGG
DKK1	TCGGTTCTCAATTCCAACGCT	GGGTACGGCTGGTAGTTGT
FOS	CGGGCTTCAACGCAGACTA	GGTCCGTGCAGAAGTCCTG
GADD45A	CCCTGATCCAGGCGTTTTG	GATCCATGTAGCGACTTTCCC
GAPDH	CCCCTGGCCAAGGTCATCCA	ACAGCCTTGGCAGCGCCAGT
GLI1	ACCCGGGGTCTCAAACTG	GGCTGACAGTATAGGCAGAGC
HES1	CTCTCTTCCCTCCGGACTCT	AGGCGCAATCCAATATGAAC
HES5	GCCCGGGGTTCTATGATATT	GAGTTCGGCCTTCACAAAAG
HEY2	GGCGTCGGGATCGGATAAATA	AAGTAGCCTTTACCCCCTGTT
MSI1	GAGGGTTCGGGTTTGTCACG	GGCGACATCACCTCCTTTGG
MYCN	CCCTGAGCGATTCAGATGAT	GACGCACAGTGATGGTGAAT
NOTCH1	CCGCAGTTGTGCTCCTGAA	ACCTTGGCGGTCTCGTAGCT
NOTCH2	ATGCTCAGCCGGGATACCT	GGTTGGCCACAGTGGTACAGG
NUMB	AGCCAGCCCATACTGCTCTA	CGGACGCTCTTAGACACCTC
PDGFRA	GAAGCTGTCAACCTGCATGA	CTTCCTTAGCACGGATCAGC
PPAP2B	CGCTCAACAACAACCCGAG	ACCAGTTTTCAGTGGGTACTTG
PTCH1	GACCGGGACTATCTGCA	GTCTGTATCATGAGTTGAGG
RP15S	TTCCGCAAGTTCACCTACC	CGGGCCGGCCATGCTTTACG
SMO	AGGCTGCACGAATGAGGTG	ACGTCCTCGTACCAGCTCTT
VEGF	CAACATCACCATGCAGATTATGC	GCTTTCGTTTTTGCCCCTTTC
WNT5B	GGGGACAACGTGGAGTACGG	AGCTGCAGCCAGCAGGTCTT

1.2. Msi1 knockdown generation

S1.a

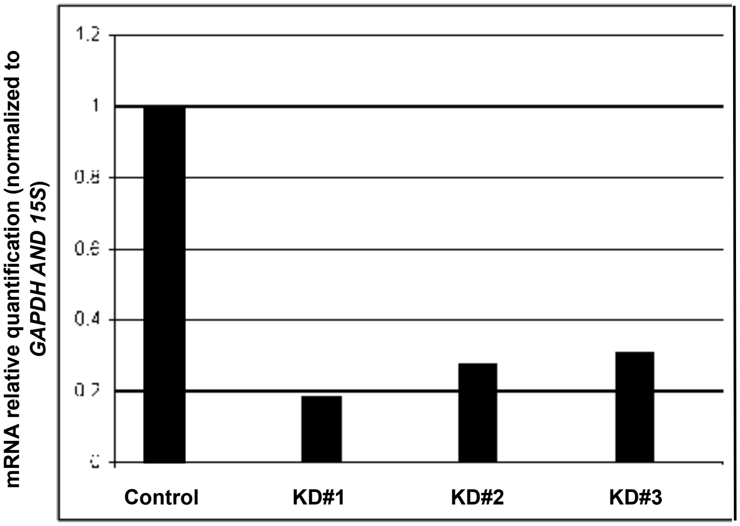

S1.b

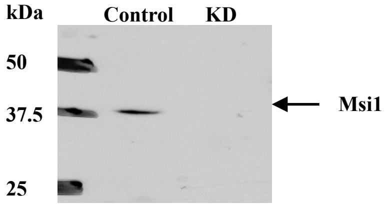

Additional file 1.

1.1. Table 1. qRT-PCR primers. The sequences of the primers utilized in this study are shown in the table.

1.2. Generation of a Msi1 knockdown in Daoy**.** Msi1 was stably knocked down in Daoy cell line using shRNA. S1.a) Three clones with at least 70% knockdown at mRNA level were selected. S1.b) Msi1 protein levels were assessed by western blot.
